# Understanding heterogeneity of treatment effect in critical care trials

**DOI:** 10.62675/2965-2774.20260021

**Published:** 2026-05-18

**Authors:** Bharath Kumar Tirupakuzhi Vijayaraghavan, Bruno Adler Maccagnan Pinheiro Besen, Leticia Kawano-Dourado, Elisa Estenssoro, John C. Marshall

**Affiliations:** 1 Apollo Hospitals Department of Critical Care Medicine Chennai India Department of Critical Care Medicine, Apollo Hospitals - Chennai, India.; 2 Universidade Federal de São Paulo Hospital São Paulo Department of Intensive Care São Paulo SP Brazil Department of Intensive Care, Hospital São Paulo, Universidade Federal de São Paulo - São Paulo (SP), Brazil.; 3 HCor-Hospital do Coração Instituto de Pesquisa São Paulo SP Brazil Instituto de Pesquisa, HCor-Hospital do Coração - São Paulo (SP), Brazil.; 4 Hospital Interzonal de Agudos General San Martin de La Plata La Plata Buenos Aires Argentina Hospital Interzonal de Agudos General San Martin de La Plata - La Plata, Buenos Aires, Argentina.; 5 University of Toronto Li Ka Shing Research Institute Toronto Canada Li Ka Shing Research Institute, University of Toronto - Toronto, Canada.

## INTRODUCTION

Heterogeneity of treatment effects (HTE) refers to the idea that the effect of an intervention varies between individuals or groups based on clinical, genetic, or other biological characteristics.^(1)^ True HTE is concerned with non-random variations in the effect of interventions. Randomized controlled trials evaluating any intervention typically provide estimates of the average treatment effect (ATE). In practice, clinicians are concerned about the effects of the treatment on the patient or individual in front of them, rather than the ATE of an intervention. For example, in a trial of an intervention that shows a beneficial effect, this may reflect a large treatment effect in a small group of individuals, with no effect or net harm in the vast majority.^(2)^ Teasing out the specific subgroups of patients that would benefit from an intervention is fundamental to our ability to tailor care and improve clinical outcomes. In measuring HTE, researchers often take one of two approaches: estimate treatment effects in clinically relevant subgroups (easier and an almost universal approach- for example, subgroup analysis by age, sex, PaO_2_:FiO_2_ ratio etc.,) or predict treatment effects for a given individual (difficult and rarely done)^(3,4)^ (Table 1).

**Table 1:** Concepts, sources, and analytical frameworks for assessing heterogeneity of treatment effects

Treatment effect descriptions	
ATE	Randomized controlled trials evaluating interventions typically provide estimates of the ATE, i.e., it is a population-level estimate of the effect of interventions
HTE	The effect of an intervention varies between individuals or groups based on clinical, genetic, or other biological characteristics.^(1)^ True HTE is concerned with non-random variations in the effect of interventions
CATE	The ATE is conditional on one or more baseline characteristics. CATE can be derived for traditional subgroup analysis up to individualized treatment effects
ITE	A type of CATE in which treatment effects are conditioned on multiple baseline characteristics. It is meant to approximate the (unobservable) true individual treatment effects
**Traditional and non-traditional sources of HTE**	
Traditional sources of HTE	Traditionally, sources of HTE investigated include demographic characteristics (e.g., age or sex) and disease characteristics (e.g., biomarkers, use of vasoactive drugs, mechanical ventilation etc.)
Other sources of HTE	Other sources of HTE that need to be considered include genetic, epigenetic, socioeconomic proximal factors, and contextual issues related to critical care delivery. These sources may hamper the transportability of HTE assessments from HICs to LMICs
**Frameworks for assessment of HTE**	
Traditional subgroup analyses	Traditional subgroup analyses are usually based on key baseline characteristics of study participants, such as age cut-offs, sex, or other factors
Subphenotype-based on subgroup analyses	Subphenotype-based subgroup analyses use the same analytical framework to assess HTE, but the "subgroups" are derived from methods that identify subphenotypes and go beyond observable baseline characteristics. Latent class analyses and cluster-based analyses are used to identify these subgroups
Traditional risk-based modelling	In critical care, the availability of prognostic models for hospital mortality enables their use in risk-based modelling (e.g., risk quantiles) to assess whether treatment effects are modified by baseline risk of death
Model-based risk modelling	Risk models may likewise be derived and validated, like effect-based models, within the dataset of clinical trials. This approach is more data-hungry than traditional risk-based modelling as it needs data both to derive and validate the model
Effect-based modelling	Effect-based modelling leverages causal machine learning with flexible models that allow interactions between treatment, multiple baseline patient characteristics and outcomes, selecting the best-fitting model to derive ITEs. This approach is data-hungry and requires large volumes of data for its derivation and validation

ATE - average treatment effect; HTE - heterogeneity of treatment effects; CATE - Conditional average treatment effect; ITE - individualized treatment effects.

In addition to variations in treatment effects driven by clinical and biological differences, the effects of an intervention are likely to depend on where it is delivered. Interventions may have differential effects across high-income countries (HICs) and low- and lower-middle-income countries (LMICs). For instance, in a *post hoc* analysis of the COVID-STEROID 2 trial^(5)^ that compared higher vs. lower doses of dexamethasone for patients hospitalized with severe COVID-19, the beneficial effects of the higher dose appeared to be much less for patients randomized in India (a LMIC) as compared to those randomized in Europe (HICs). Similarly, in the Anticoagulation Strategies in Non-Critically Ill Patients with COVID-19 trial^(6)^ that compared low, intermediate, and therapeutic doses of anticoagulation and enrolled over 90% of the patients from India and Nepal (LMICs), intermediate dose anticoagulation had the highest probability of a favourable effect. This finding contrasted with the results of a multiplatform anticoagulation trial that enrolled patients predominantly from HICs and suggested that therapeutic-dose anticoagulation was beneficial.^(7)^ These differences in the effect of an intervention by geography are likely a consequence of differences in biology and also potentially a reflection of differences in culture, health systems, and resource availability. Importantly, the presence of HTE, whether at the patient or geographical level, has implications for our ability to translate the results of randomized controlled trials.

## CURRENT RESEARCH GAPS AND OPPORTUNITIES

Traditionally based on subgroup analyses of baseline categorical variables, HTE assessment has undergone recent developments, including risk-based modelling^(2)^ and effect-based modelling.^(4)^ Additional methodological approaches have been proposed, such as the Predictive Approaches to Treatment effect Heterogeneity (PATH) statement,^(8)^ to incorporate both risk- and effect-based modelling in HTE assessment.

Two main gaps from such assessments of HTE persist: how they are transported to other settings, as they have been conducted mostly in studies from well-resourced settings in HICs; and how effect-based modelling may be applied in practice, as these analyses have been conducted post hoc without external validation in real-world settings.

These gaps represent important opportunities for further research. Additionally, even modern methods of HTE assessment rely on baseline variables from clinical trials, which do not take advantage of the unique opportunity to explore other aspects that may lead to HTE beyond past comorbidities, acute physiology, and presentation characteristics. For example, genetic variability, differential practice approaches, and a spectrum of more proximal socioeconomic factors may also lead to unexplained variation in treatment effects.

Clinical trials offer an important opportunity to explore HTE, provided that these analyses are planned. In identifying systemic or contextual sources of HTE, large multinational trials not only enhance study power but can also reveal variability in treatment responses contingent on the socioeconomic characteristics of recruiting sites. Randomization within a trial is a powerful tool for inferring causality. So, the routine collection of biological samples during a trial enables post hoc studies to identify biological variables associated with differential treatment response. In addition, the use of adaptive designs in clinical trials enables the employment of biomarker enrichment strategies, shifting randomization proportions towards populations identifiable by a biomarker that experience a greater response to treatment.

## LIMITATIONS, CHALLENGES, AND MITIGATION STRATEGIES

Assessment of heterogeneity of treatment effect in clinical trials has two main limitations. First, such strategies suffer from limited power to derive meaningful estimates, whether they are traditional subgroup analyses or risk- or effect-based modelling analyses, both of which are increasingly data-hungry. Second, and very relevant for knowledge translation between different contexts, transportability of findings from well-resourced settings to low-resource settings may not be straightforward, as contextual issues related to the structure and process of care of critically ill patients in low- and middle-income countries, among other issues, may hamper applicability of findings to guide clinical decision-making.

The main challenge, therefore, is how to assess and transport HTE findings between different contexts. Another challenge to be addressed is underfunding for statistical expertise in LMICs, which would be needed to perform more complex analyses, such as cluster analysis for subgroup analyses and risk- and effect-based modelling.

These challenges may be mitigated through some strategies. First, there is a need to validate HTE assessments across different contexts. Second, the deployment of large, pragmatic trials may facilitate accrual to larger sample sizes, enabling more powerful analyses for HTE, but at the expense of explainability. Third, registry-embedded strategies may be an option both in registry-embedded clinical trials that reduce the burden of data collection and leverage their availability even in LMICs, and in modern methods of causal inference through target trial approaches that can fit within the frameworks for HTE assessment in clinical trials.

However, registries are inherently limited by their observational nature, which can introduce confounding by indication and various forms of collider bias, leading to noise in assessments. Additionally, although granular data may be available at baseline and for some interventions during an ICU stay and for hospital outcomes, data on important prognostic variables to be tested may not be available and may require additional data collection. Registries are not fundamentally designed to test heterogeneity of treatment effects, and as a result, the ability to assess HTE using registry data across a variety of interventions is currently limited.

It is also important to highlight other fundamental limitations of HTE analyses. Such analyses undertaken in the absence of a biological rationale, when not pre-specified, and when purely data-driven, are prone to spurious findings and misinterpretations. An extreme, but well-known and cited, example that illustrates the problems of ill-conceived HTE analysis is the analysis of aspirin subgroup effects by zodiac sign in the Second International Study of Infarct Survival (ISIS-2).^(9,10)^ To mitigate such problems, researchers have recently proposed the Instrument to assess the Credibility of Effect Modification ANalyses (ICEMAN) in randomized controlled trials and meta-analyses.^(11)^ The tool includes a number of elements; whether the direction of effect modification was correctly prespecified a priori, whether it was supported by prior evidence, whether a test for interaction suggests that chance is an unlikely explanation, and so on. Researchers considering an HTE analysis would be well advised to review the ICEMAN tool before embarking on multiple subgroup analyses.

## RESEARCH ROADMAP

As modern strategies to assess HTE require even more data to derive meaningful estimates, registry data, with their large volumes, may overcome these limitations. Additionally, registries exhibit contextual and geographical diversity, which may enable evaluations of transportability across different settings, making knowledge translation more feasible.^(12)^

In practice, future research on HTE in clinical trials should leverage ICU registries through the deployment of registry-based RCTs, which would allow inclusion of large enough samples of participants from different contexts, perhaps without the need for additional data collection. Alternatively, conducting target-trial emulation studies embedded within registries, with additional data collection of pre-specified variables to properly emulate the trial, is another possible solution to leverage their potential for causal discovery^(13)^ and to move beyond prognostic studies. However, the feasibility of this approach has yet to be consistently demonstrated.

Finally, machine learning-enabled HTE^(14)^ may offer a more feasible and equitable strategy than traditional approaches. Classical HTE methods require extensive local analytic capacity and bespoke modelling that many LMIC health systems cannot support. In contrast, ML-based individualized HTE models can be trained centrally, validated externally, and deployed widely at low marginal cost, allowing LMICs to access individualized predictions without the need for large local datasets or specialized biostatistical expertise (Table 1).

## Data Availability

The contents are already available.
